# Defining Collective Identities in Technopolitical Interaction Networks

**DOI:** 10.3389/fpsyg.2020.01549

**Published:** 2020-07-31

**Authors:** Xabier E. Barandiaran, Antonio Calleja-López, Emanuele Cozzo

**Affiliations:** ^1^IAS-Research Centre for Life, Mind, and Society, Department of Philosophy, Faculty of Labour Relations and Social Work, University of the Basque Country (UPV/EHU), Vitoria-Gasteiz, Spain; ^2^Decidim Research, Laboratori d’Innovació Democratica, Barcelona, Spain; ^3^Internet Interdisciplinary Institute (IN3), Universitat Oberta de Catalunya, Barcelona, Spain; ^4^Institute of Complex Systems (UBICS), Universitat de Barcelona, Barcelona, Spain

**Keywords:** collective identity, social identity, social interaction, digital networks, social network analysis, technopolitics

## Abstract

We are currently witnessing the emergence of new forms of collective identities and a redefinition of the old ones through networked digital interactions, and these can be explicitly measured and analyzed. We distinguish between three major trends on the development of the concept of identity in the social realm: (1) an essentialist sense (based on conditions and properties shared by members of a group), (2) a representational or ideational sense (based on the application of categories by oneself or others), and (3) a relational and interactional sense (based on interaction processes between actors and their environments). The interactional approach aligns with current empirical and methodological progress in social network analysis. Moreover, it has been argued that, within the network society, the notion of collective identity (Melucci, 1995) in the political field must be rethought as technologically mediated and interactive. We suggest that collective identities should be understood as *recurrent*, *cohesive*, and *coordinated communicative interaction networks.* We here propose that such identities can be depicted by: (a) mapping and filtering a relevant interaction network, (b) delimiting a set of communities, (c) determining the strongly connected component(s) of such communities (the core identity) in a directed graph, and (d) defining the identity audiences and sources within the community. This technical graph–theoretical characterization is explained and justified in detail through a toy model and applied to three empirical case studies to characterize political identities in party politics (communicative interaction in Twitter during the Spanish elections in 2018), contentious politics in confrontation (in Twitter during the Catalan strike for independence 2019), and the multitudinous identity of Spanish Indignados/15 social movement (in Facebook fan pages 2011). We discuss how the proposed definition is useful to delimit and characterize the internal structure of collective identities in technopolitical interaction networks, and we suggest how the proposed methods can be improved and complemented with other approaches. We finally draw the theoretical implications of understanding collective identities as emerging from interaction networks in a progressive platformization of social interactions in a digital world.

## Introduction

The use of the notion of identity, especially in human and social sciences, has skyrocketed from the 1960s onward, so much so that some authors have denounced the overextension and misuse of the concept (Polletta and Jasper, 2001), while others have called for its abandonment and replacement with other more concrete ones (Brubaker and Cooper, 2000). We believe that the role of the concept in personal and social life (both implicitly and explicitly), as well as its strong role as a currency across academic fields, especially in the social sciences, suggests that discarding it is nowadays both practically and epistemically unproductive. We believe instead that a work of systematization, operationalization, and update of the notion of identity in the social sciences is needed. Building upon a previous work (Monterde et al., 2015), in the present paper, we try to advance in that direction. We do so with the case of collective identity, a relevant notion in social and political theory as well as in areas of research such as social movement studies (Melucci, 1989; Gerbaudo and Treré, 2015) and social psychology (Simon and Klandermans, 2001).

In this paper, we provide an operational definition of collective identities as emerging from interaction networks. The set of analytic tools provided here embodies conceptual and theoretical assumptions that are critical to the definition and understanding of collective identities. In turn, pragmatically understood, collective identities are, we believe, defined by the tools used to study them. This is why the present paper brings together abstract sociological discussions and detailed technical specifications. As a mapping of sorts, we first review the sociological literature, focusing on interactive conceptions of social identity. Afterward, we articulate a discussion of digitally mediated collective identities, especially in the field of politics. We then examine what network theory has to offer to characterize them. We propose that structural and dynamic formal aspects of such identities can be depicted by: (a) mapping and filtering a relevant interaction network, (b) delimiting a set of communities, (c) determining the strongly connected component(s) of such communities (the core identity), (d) defining the identity audiences and sources within the community, and (e) analyzing the identity collective cohesion of the identity core and its nested internal structure. This technical graph–theoretical characterization is explained and justified, illustrated with a toy model, and applied to three case studies: (a) political-party identity groups during the Spanish general elections in 2019 on Twitter, (b) identity confrontation on Twitter during a general strike against the trial of the Spanish State against Catalonian politicians, and (c) Facebook fan page interactions within the 15M/Indignados^1^ social movement. We finally discuss some of the implications of our definition, how it relates to the different theoretical approaches to understand collective identities, how it can be extended and improved with various methods, and how it might gain relevance not only as an analytic contribution but also as a synthetic device in the technopolitical context of an ever-growing digital platformization of the public sphere.

## Mapping (Collective) Identity: a Brief and Broad Approximation

Identity has been a popular concept in the social sciences since the 1960s^2^. Core to such popularization is the work of Erikson (1968), who understood identity as a process of bidirectional identification between individual and community. Also in the 1960s, the rise of the Black Power movement (a template for later identity movements), along with the weakness of left institutions and class discourse, facilitated the rise of identity language. In the 1980s, the rise accelerated with the emergence of cultural studies and its emphasis on race, gender, and class and their relation to identity. Social movements such as LGTBI also contributed to the political and the social spreading of the notion.

The notion of collective identity is nowadays central to sociological theorizing. It came to fill the gaps left by existing theories of social organization (Polletta and Jasper, 2001). It gained momentum in order to account for how phenomena such as social movements could display a consistent collective behavior despite a lack of strong institutional incentives (economic, hierarchical, legal, or otherwise). Melucci’s (1989, 1995, 1996) writings are now the obligatory entry point to the literature on collective identity, which we discuss in the following section.

Before exploring the debate opened by his work, it is important to analytically distinguish between the three senses or dimensions of *social grouping or identity* that we have found in the literature in social sciences: (1) an essentialist sense (based on properties or conditions shared by members of a group), (2) a representational or ideational sense (based on the external application or self-application of categories and representations), and (3) a relational or interactional sense (based on interactive relations between actors or between actors and their environments). Not infrequently, positions mix these aspects. Using this threefold context, we situate now some central positions in the social sciences that will help to situate our approach in that broad landscape.

A first approach, we named “essentialist,” frequently tries to reveal social groups by looking at (what are taken as) objective conditions or traits that are shared among the members of that group (material socio-economic conditions, genetic properties, sexual orientation, linguistic competence, or historical traits). An example of the attention to the objective dimension of identity in sociological analysis is that of orthodox, economy-centric class analysis (Wright, 2005)^3^, but many paradigmatic cases involve biological, psychological, or cultural traits (Sayer, 1997).

A second approach has stressed the primacy of *ideational or representational elements* in the shaping of social groups. Classical theories of social identity attend to processes of social categorization and identification. From Tajfel and Turner (1979) to Anderson (2016), from social constructivism (Berger and Luckmann, 1967) to gender theory (Butler, 1990; Lorber and Farrell, 1991), the application to others and to oneself of concrete discursive categories and representations has become central in discussions on identity. Constructivist approaches have suggested that discourse helps to construct the groups that, from a positivist standpoint, it allegedly describes [for a classical example in political theory, see Laclau and Mouffe (1985), Laclau (2005)].

Finally, a third approach attends to social relations and, especially, interactions as the basis of collective identity. The *New York School of Relational Sociology* “affords primacy, both ontological and methodological, to interactions, social ties (“relations”), and networks” (Crossley, 2015, p. 66). This tradition has often been associated to that of social network analysis [SNA hereafter; see Scott (1988)] in the last decades (Crossley, 2015), but it has also been differentiated from it, sometimes opposed as “theories of networks” vs. “network theory” (Borgatti and Lopez-Kidwell, 2014) or “relationism” vs. “formalism” (Erikson, 2013). While the former would attend to practices, culture, meaning, agency, and contingency, the latter would attend to mathematics, structure, formality, universality, etc. Despite their differences, both approaches should be regarded as complementary within a unified interest on reconstructing social categories on the basis of relations and interactions. Regarding the issue of collective or social identity, on the relational sociology side, Charles Tilly has proposed that “interpersonal transactions”^4^ are “the basic stuff of social processes […], compound into identities, create and transform social boundaries, and accumulate into durable ties” (Tilly, 2006, p. 6). On the formalist side, however, little attention has been paid directly to the concept of collective identity, but considerable progress (both formal and empirical) has been made on the understanding of social solidarity, group formation, and social cohesion and the way in which embeddedness in interaction structures gives rise to processes of identifications. So, for instance, Moody and White (2003) have shown how interaction network structural properties explain “ideational components of solidarity in a dozen large networks” of adolescent friendships and their “identification” with school.

We believe that both relationism and formalism, in their common emphasis on interactions, are key to rethink the social context today. In the present work, we rely more on the formalist SNA tradition. The availability of interaction data, the exponential increase in computational capacity for analysis, and the theoretical maturity of the graph theory provide an empowering methodological context for formal approaches that can now be integrated in the emerging field of computational social sciences (Lazer et al., 2009). However, the situation is not only methodologically favorable. As we are about to see, the increasing predominance of digital or technopolitical interaction networks on the formation and the maintenance of collective identities makes formal and SNA approaches more socially relevant today.

### Collective Identities: An Open Debate From Social Movements to Systems Theory

Melucci’s (1989, 1995, 1996) proposal of the notion of collective identity tried to bring attention to aspects of collective action and social movements neglected by previous approaches: frequently informal, emotional, and cultural aspects—and, ultimately, identity—were thereby brought to the fore at every level of analysis (Polletta and Jasper, 2001; Snow, 2001; Opp, 2009; Flesher Fominaya, 2010). Under the ideational or representational paradigm, research on frame theory (Snow and Benford, 2000) connected with many of these leitmotivs and provided new tools for understanding how collective actors construct their shared views, motivations, and feelings about themselves and the world.

As we have discussed in an earlier paper (Monterde et al., 2015), to go beyond the slipperiness (Flesher Fominaya, 2010) and overextension (Polletta and Jasper, 2001) of the concept of collective identity in the literature, what may be required is a clear definition, systematization, and operationalization of its various aspects. As we noticed there, too, Snow (2001) has rightly shown that collective identities can be multidimensional—including cognitive, emotional, and moral dimensions (Melucci, 1989). In that work, we showed that attending to the interactional dimension (beyond ideational or representational approaches such as frame theory) was required and that such an interactional approach required, in turn, a network approach. However, we did not provide a proper and rigorous definition of collective identity that could be applied to other case studies.

Interestingly, Melucci gave a system- and network-friendly definition of collective identity by considering it as “a network of active relationships between the actors, who interact, communicate, influence each other, negotiate and make decisions. Forms of organization and models of leadership, communicative channels and technologies of communication are constitutive parts of this network of relationships” (Melucci, 1995, pp. 44–45).

**FIGURE 1:**
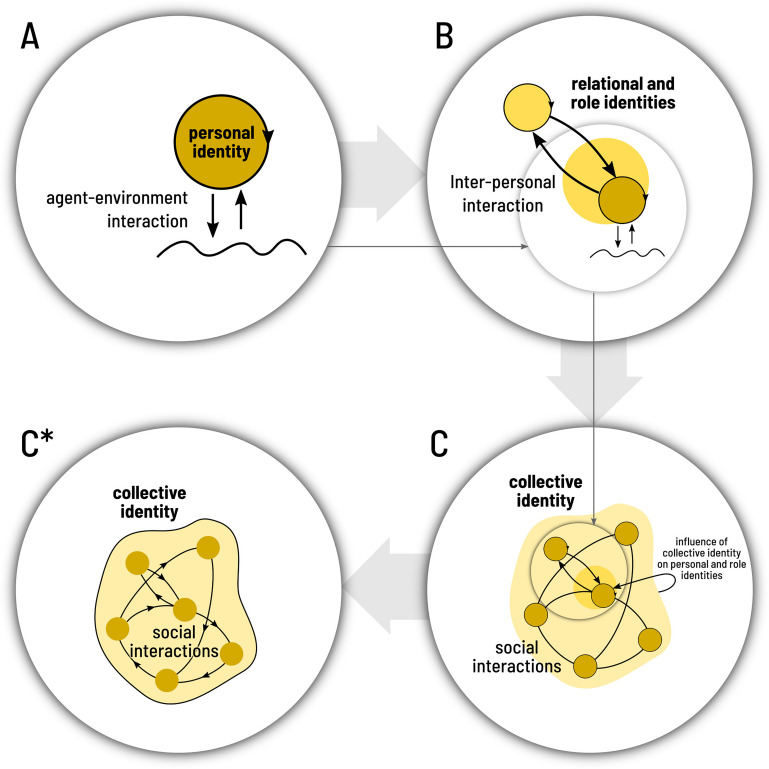
Different scales of social identity, from the personal to the collective. Circles indicate individual agents, arrows indicate not only a direct interaction but also, more importantly, a modulation of the interactions (for simplicity, we have avoided drawing arrows over arrows). For simplicity, we also abstract away the internal network of interactions that gives rise to individual identity. **(A)** The way in which personal identity is built in interaction with the environment. **(B)** Other agents on the environment and how personal identity is thus shaped through interpersonal interactions that create relational and role identities in the process. **(C)** How a network of social interactions gives rise to an emergent collective identity; in turn, this identity affects the personal identity. In this article, we focus exclusively on how a collective identity emerges from a network of social interactions, bracketing; for the purpose of this analysis, the complexities involved in the interaction between different scales of personal and social identity. The simplified final scheme is illustrated in panel **(C^∗^)**.

From the complex systems tradition, what is crucial for the emergence of identities are the interactions between the elements of a system (Sawyer, 2005), between that system (or some of its parts) and its environment, and between that system and itself (in first-, second-, and third-order relations). The relationship between personal–psychological identity and social collective identities is complex and multifaceted (Stets and Burke, 2000). Since our task is to define the identity of collective identities, here, we are simply going to outline a basic understanding of their emergence in order to properly isolate and delimit our proposal for collective identity. Figure 1 shows this process of abstraction and the scale of analysis that we will focus on.

### Collective Identity and the Politics of the Network Society: Varieties of Technopolitical Inter-Identities

From the path-breaking work of Castells (1996) onward, a growing body of research has shown the social transformations associated to (not determined by) the extension of digital technologies into an increasing number of activities and spheres, from economic to political. Promoted by a variety of actors, from governments and corporations to individual and organized citizens, this extension has crucially shifted the modes of information and communication and, in relation to them, the forms of constructing social phenomena such as collective identity, organization, action, power, culture, or politics (Kellner, 1999; Chadwick and Howard, 2008; Castells, 2009, 2012; Earl and Kimport, 2011; Bennett and Segerberg, 2012; Bennett et al., 2014; Coleman and Freelon, 2015; Gerbaudo and Treré, 2015). Technologies facilitate new forms of interaction (which also redefine those technologies), thereby bringing about new forms of identity (which, in turn, affects the other two).

Information and communication technologies and practices around them are at the core of such transformations. In our analysis, we look at cases from the field of politics, more specifically, party politics (Katz and Crotty, 2006) and contentious politics (Tilly and Tarrow, 2006). For that, we believe that the notions of *technopolitics and technopolitical interactions are key*. In a synthetic fashion, Hecht (2009, pp. 56–57) has defined technopolitics as “the strategic practice of designing or using technology to constitute, embody, or enact political goals.” It points toward both the technological construction of politics and the political construction of technology. This double direction of the relation between technology and politics is fundamental in order to understand the new forms of collective identity emerging in the network society.

Throughout the twentieth century, mass media were crucial in the shaping of politics as well as individual and collective identities. According to Douglas Kellner, the difference of recent technopolitics resides in the possibilities afforded by the web for things such as instantaneous worldwide communication, increased multimedia interactivity, archived discussion, and, more importantly, moving from a one-to-many broadcasting model of communication toward a “computer-mediated communication [that] is highly decentralized and makes possible many-to-many communication” (Kellner, 1999, p. 103). This means that, against traditional mass communication controlled by the State or big media corporations and usually reflecting elites’ views (be those of the owners, managers, or sponsors), web-based “political communication is more decentered and varied in its origins, scope, and effects” (Kellner, 1999). Castells (2009) has built upon this intuition about the many-to-many communicative structure enabled by the Internet and, later, social media. This generates a phenomenon which he defines as *mass self-communication*: “mass communication because it can potentially reach a global audience […] it is self-communication because the production of the message is self-generated, the definition of the potential receiver(s) is self-directed, and the retrieval of specific messages or content from the World Wide Web and electronic communication networks is self-selected” (Castells, 2009, p. 55).

Although we find much value in Castells’ notion of mass self-communication, we believe that something else is going on, in relation to collective selves or identities and contemporary technopolitics. New forms of communication in contentious politics and in social movements, such as 15M/Indignados, demand to rethink social identity not only in terms of masses but also of multitudes. Hardt and Negri (2004) have distinguished the mass as an internally undifferentiated and inert aggregate of people from the multitude as a collective “composed of a set of singularities… whose difference cannot be reduced to sameness” (Hardt and Negri, 2004, p. 99). As we have shown, at the core of movements such as 15M/Indignados, there were *multitudinous identities*, that is, internally complex, decentralized, diverse, multipolar, digitally mediated, and collective identities (Monterde et al., 2015). In view of these factors, it might be more appropriate to speak of *multitudinous self-communication* (Calleja-López, 2017) rather than of mass self-communication for some new emerging cases of collective identities, like the 15M movement.

Today party politics can combine dynamics of mass and multitudinous self-communication with various forms of automated politics. Campaigns include processes of political automation: the use of chatbots, posting bots, false profiles, and the automated inflation of metrics and followers (Bessi and Ferrara, 2016). They are frequently tied to the diffusion of fake news: biased, incomplete, or spurious media stories with exaggerated and emotional adjectivation (Graves, 2018). Finally, there are strategic communication companies, such as Cambridge Analytica, who have intervened in the last presidential campaigns of the US, Argentina, Mexico, Brazil, Sri Lanka, Malaysia, China, Australia, and South Africa, as well as the referendum that caused the separation of Britain from the European Union. These cases have drawn public attention to the use of these platforms for influencing and shaping public discourse and action (Tufekci, 2018) or to the emergence of alt-right collective identity (Garpvall, 2017; Gray, 2018), where the use of bots and algorithmic tactics seems to have played a prominent role (Daniels, 2018). In synthesis, there has been a rise of what some have defined as “datapolitik” or datacracy, which points to the strategic use of big data and digital platforms to gain and exercise political and cultural power (Gambetta, 2018).

These digitally networked practices and dynamics become more and more prominent, transforming much party and contentious politics into *party* and *contentious technopolitics* (Calleja-López, 2017). To think of collective identities today, it is necessary to build a technopolitical and an interactional approach to them. To build such an approach and to apply it to three different cases is the task of the following sections.

## Proposal: Collective Identities as Strongly Connected Cores Within Communities and Environments in Digital Interaction Networks

### Introduction, First Approximation

The concept of “operational closure” has been used in complex system approaches to biological and cognitive systems to characterize the emergence of identities in interaction networks (Varela, 1979; Barandiaran et al., 2009). In the realm of the origins and emergence of life and cellular biology, the identity of the living is characterized as emerging from metabolic molecular interaction networks (Maturana and Varela, 1980; Ruiz-Mirazo et al., 2004). Recent progress in embodied and enactive psychology also conceives of identity as emerging from networks of behavioral and neural interactions (Thompson and Varela, 2001; Di Paolo et al., 2017). Moreover, the case has been empirically made that cognitive and psychological processes are interaction-dominant (Ihlen and Vereijken, 2010), meaning that the nature of the cognitive process lies at the interaction between components and not at the decomposable functioning of any such components (neurons, muscles, tools, etc.). These characterizations of identity rely on the concept of operationally closed systems understood as those that “(i) continuously regenerate and realize the network that produces them, and (ii) constitute the system as a distinguishable unity in the domain in which they exist” (Varela, 1997, p. 76). Although this and previous definitions (Maturana, 1970) have also been used to characterize social systems (Luhmann, 1986, 1995), there has been (to our knowledge) no previous application to characterize collective identities, neither has an empirical application of this approach in social media been attempted before.

Following the aforementioned tradition, we provide a first operational (formalist and interactional) characterization as follows: *a collective identity is the interaction network that is both the result and the source of recurrent, cohesive, and coordinated communicative interactions between different agents across different communication spaces, distinguishing itself from the environment and other identities within a communication scope*. The collective identity is sustained and defined by the network of interactions between individuals and between the resulting system and its environment. From this network, collective claims emerge, define its boundaries, and reinforce the interaction network itself. The exclusiveness of an identity will depend on the polarizing conflicts that tear it apart from others. The strength of a collective identity is determined by the degree of interactive integration or embeddedness of individuals.

### Operational Characterization

**FIGURE 2:**
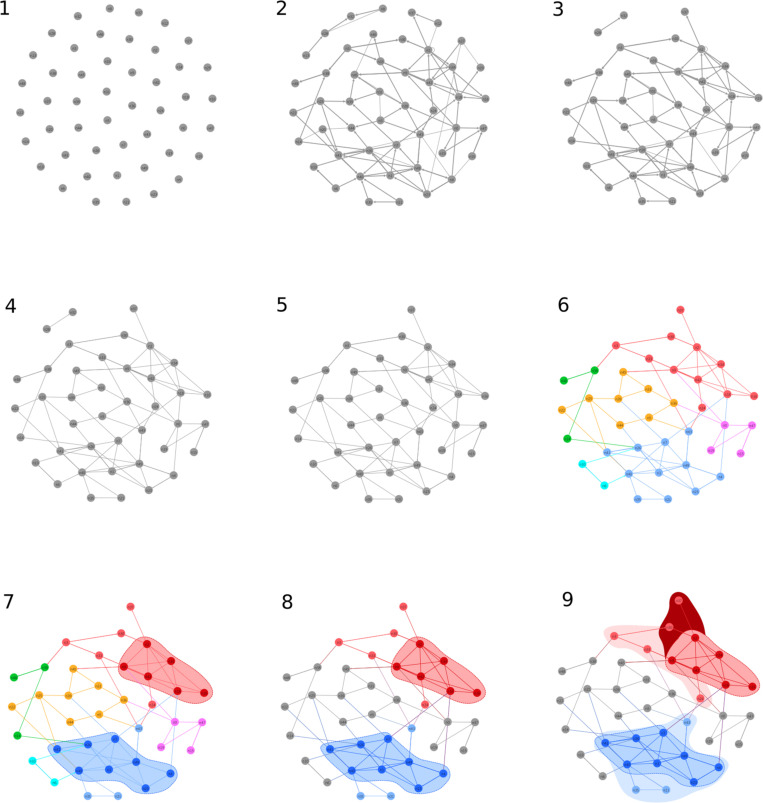
Conceptual and algorithmic steps to specify collective identities in a social interaction network: **(1)** collection of individuals, **(2)** set of interactions in a specific interaction space, **(3)** filters a scope within the space, **(4)** interactions are filtered, **(5)** a giant connected component is isolated, **(6)** communities are identified, **(7)** the strongly connected components of the communities are identified, **(8)** those modular or community partitions without identities are turned into environment, and **(9)** core, audience, and identity sources are distinguished within communities (see text for further details and see Figure 3 for details of subfigure 2.9).

**FIGURE 3:**
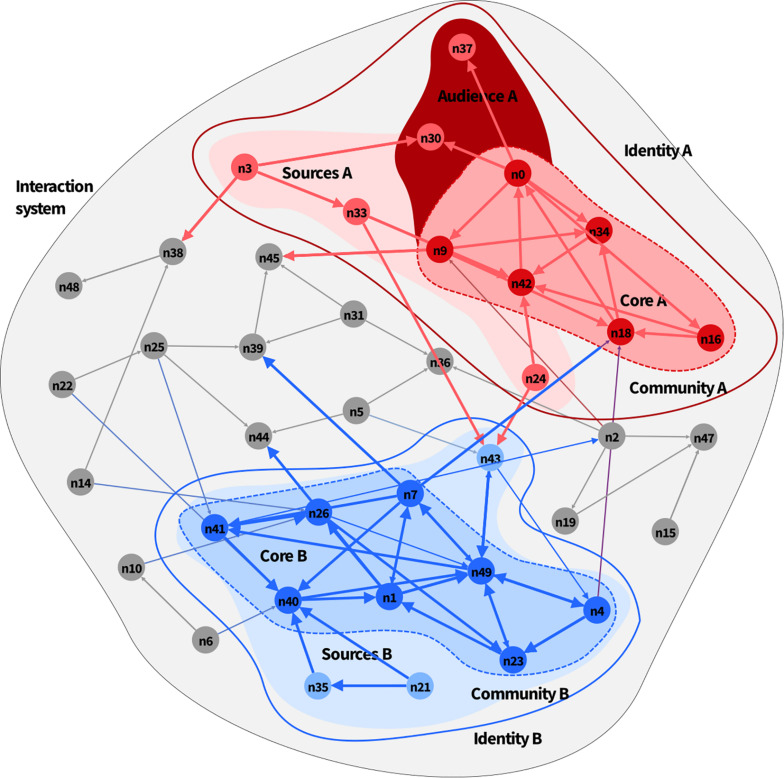
Complete analysis of the interactive identities in a system: Two identities, (A,B), coexist within the system with a set of nodes being their environment (in gray). Each core identity inhabits a community made of sources and an audience.

**FIGURE 4:**
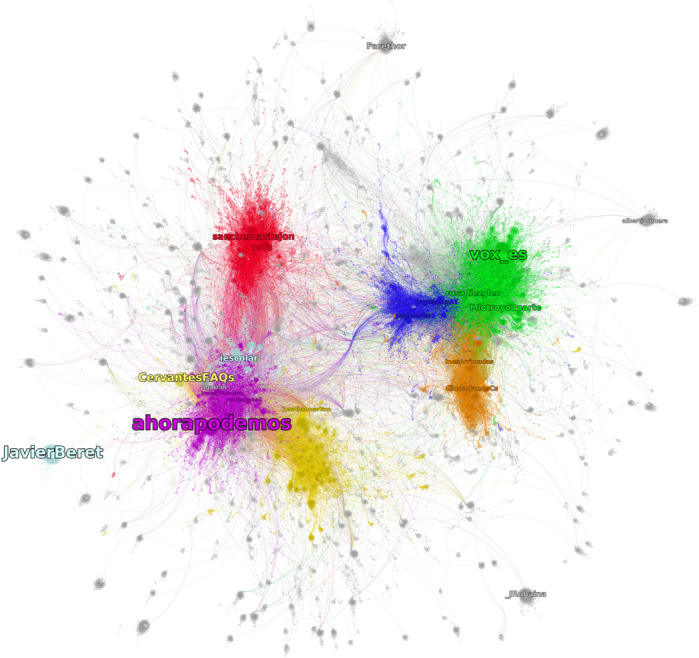
Nodes are twitter handles, links represent retweets, and the direction of the link is indicated by the curvation, with the direction being aligned in a clockwise direction. The colors represent communities. Gray communities are those without an identity core. Light blue and light yellow communities are without core but formed by the news media and political commentators.

**FIGURE 5:**
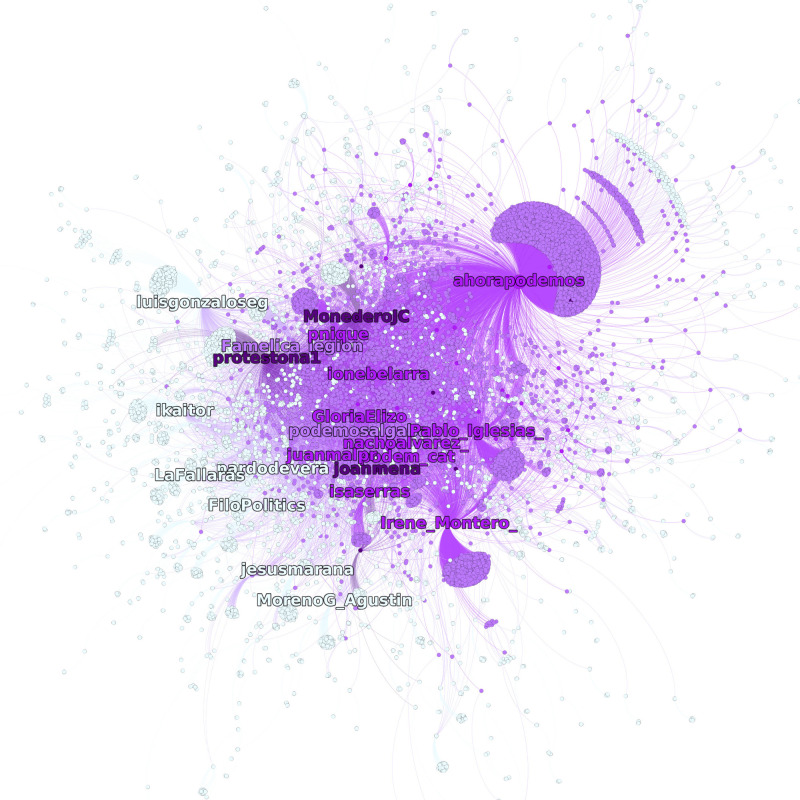
Podemos’ community. In purple, the nodes in the identity core; in light purple, the audience; and in dark purple, the sources. In light blue, the nodes that are not directly connected to the identity core.

We define a network-theoretical characterization of social and political collective identities based on (technical terms and in italics and will be explained below):

(1) Scope specificity and space multiplicity (steps 1–3 in Figure 2);

(2) Interaction significance (*filter*) (step 4 in Figure 2);
(3) Systemic connectedness (*weak connected component*) and community integration (*modularity*) (steps 5 and 6 in Figure 2);
(4) Identity core(s): closure to interaction coordination (*strongly connected component* and *k-components*) in directed graphs (steps 7 and 8 in Figure 2);
(5) Identity audiences and identity sources (step 9 in Figure 2).

The final result can be illustrated in Figure 3. We shall now move step by step in explaining the underlying assumptions, justifying the algorithms and analytical tools, and making explicit references to the toy model illustrated in Figures 2, 3 before we move to real-case scenarios. Although some steps or procedures might seem to be purely technical, they nevertheless embody important conceptual assumptions. In operational terms, the specification of a method to characterize identities is both a methodological and a conceptual process. We therefore detail the whole method in the following pages.

#### Scope, Space, and Agents

##### Collective identities belong to interaction scopes across interaction spaces between individual agents

We understand by interaction *space* the medium or mode of structured interactions between persons, which can be social media such as social network (Twitter and Facebook) or physical places such as a room and also activities or practices such as voting, shopping, etc. In the present paper, we analyze a type of space where interactions take primarily (although not exclusively) the form of communication^5^. In such a (communicative) space, it is possible to distinguish different *interaction scopes*, that is, different thematic spaces or topics of communication, e.g., gender, sports, politics, etc. Usually, the same interaction scope cuts across different interaction spaces: e.g., a political topic is built cutting across campaign meetings, Twitter, street posters, televised debates, etc. Similarly, the same space hosts different interaction scopes: e.g., Twitter can accommodate simultaneously communicative interactions about football, politics, and gender at the same time and among the same individuals. Finally, *individual agents* are defined as nodes of communication that hold a specific identifier or reference on the communication space (e.g., a username). Note that they need not be humans nor unique. When formalized or visualized as a network, individual agents are pictured as a node that can be controlled by an autonomous computer program, a human or multiple humans, or a combination of them. We use the adjective individual to point out the indivisible nature of their display (you cannot divide or split a social network login account into two) and to distinguish it from potential *collective* agencies that would emerge from the interaction between individual agents^6^.

#### Interaction Significance

##### Collective identities are structured sets of significant interactions between agents

Once the space or spaces of observation, the individuals, and the scope or scopes are determined, it is still necessary to specify what counts as a proper interaction. When interactions are not digitized and directly recorded, the problem arises as to what the threshold is to consider a measurable variation relevant (what to record). Once recorded, the question still remains as to whether a specific interaction is relevant for outlining identity.

In such cases in which interactions are cumulatives/countables (e.g., retweets, phone calls, etc.), the network representations of the system is a weighted network, i.e., the link representing the interactions between two nodes has an associated weight (e.g., number of retweets, number of phone calls, etc.). As a result, non-significant interactions can be filtered out mainly in two ways: (a) by fixing a global threshold and retaining only the interactions that exceed the threshold and (b) by retaining all the interactions that locally, i.*e*., at the level of the node, carry a disproportionate fraction of the total weight of the interactions emanating from that node (e.g., if an agent talks to 10 people in one day but in two cases the conversation lasts more than 5 min and the remaining eight conversations are only a few-second “hey” or “good morning”; these last ones are removed and the rest was retained). It is known from different empirical analysis that global thresholding will result in a filtered network whose topological properties may be very different from the original network. Type b filters, because of their local nature, retain much more information instead (Serrano et al., 2009; Sagarra et al., 2014)^7^.

#### Systemic Connectedness and Community Integration

##### Collective identities exist within interaction systems and within communities that are more internally connected than they are with the rest of the network

We define an interaction (eco)system as the *giant weakly connected component* or the *biggest connected subgraph*. Simply put, the interaction system is the network of interactions that connects all the individual agents. After cutting out a subset of all types of social interactions, the subset defined by a given scope and, having filtered out the insignificant or irrelevant interactions, the resulting network might be split into two or more subnetworks. This is the case in Figure 2 (sub Figures 2–4): Within the space and the scope, the whole system is split into two, with a small subnetwork on the top-left side and a giant component. So, the first step is to isolate an interaction system and find the identities within, but before we move into finding the core identities, we first need to find their interaction communities, also called modules in network theory. An interaction community is a *cluster of agents that interact more between themselves than what they do with the rest of the environment*.

From the point of view of the collective identity (still to be characterized) of this module or community: it is the most proximal recurrent interaction environment for the identity core and can be distinguished from the rest of the network environment. The fixation of these communities is, to some extent, relatively dependent on a set of parameters that might split a given network or more or less (smaller or larger) communities. Knowledge of the systems and the expected communities and their boundaries is often required to fix such a parameter to deliver “the right” split of the network. This is often inevitable and shows one of the limits of interaction-centered structural analysis with incomplete information. It is also a result of the nature of identities: that they frequently appear nested (one can be an activist, leftist, socialist, and anarchist) and that there is no single privileged scale of collective identity construction that can be structurally identified.

Community detection algorithms or modularity algorithms split the whole network into communities or modules, leaving no nodes out of the partitions (Fortunato and Hric, 2016), and yet not every agent belongs to a collective identity or not necessarily. It is often the case that some networks split into communities that are the habitat of a given collective identity and other “communities” are simply the more general environment of the communicative ecosystem without giving birth to collective identities. In other words, a partition of the whole interaction system into those clusters of nodes that have more internal ties than they do with the rest of nodes does not mean that all those partitions are themselves identities. Thus, we need to move to the next step on identifying collective identities in order to clarify which of the partitions are properly communities for collective identities and which ones are not but are instead simply part of an unidentified environment.

#### Identity Core(s): Closure to Interaction Coordination

##### An identity core is the strongly connected component of the community

It is time to identify the core of an identity. We have defended that recurrent, cohesive, and coordinated communicative interactions define a collective identity. What identifies this core is its closure to interaction coordination: the property by which nodes of a subnetwork reciprocally influence each other in an effective manner and nodes that externally influence the subnetwork are not in turn influenced by members of the subnetwork nor external nodes that are influenced by subnetwork members influence back. We defend that these properties translate, within a directed functional network structure, into the notion of a *strongly connected component* and its *k-cores*. The canonical definition of a strongly connected component is as follows: “A digraph D is strongly connected or strong if each point is reachable from each other point” (Harary, 1967, p. 18), meaning that given any *i* and *j* within the graph, there exists a directed path from *i* to *j*. A node *i* is globally reachable if, for every other node *j*, there exists a directed path in G from node *j* to node *i*. In turn, C is a *strongly connected component* of a given network N if C is strongly connected and there is no strongly connected component in N that contains C.

The concept of a strongly connected component is only applicable to directed graphs (like Twitter or Facebook “like” connections), that is, in networks with arrows where the information flow or the dynamic influence or causality between variables is directed^8^. Information (or influence) can circulate within a strongly connected component, potentially departing from and reaching any node of the component. Note also that we are analyzing *significant interaction* networks within relevant *scopes*, that is, we are not analyzing mere relations like A being a friend or follower of B and, therefore, potentially receiving information from B. For a directed link to exist between B → A, it is necessary that A mentions or retweets or likes B’s message, that is, a real communicative interaction needs to occur, and this has to be *significant* (in the context of the overall communication intensity and compared to a random distribution of interactions) and that it occurs in a specific scope (thus, ruling out effective and repeated communications that are nevertheless trivial, like saying “good morning” on the lift or “today is finally Friday!” on Twitter).

It is reasonable to assume that actors (or nodes) A and B are part of the same collective identity if they are both influenced by and, in turn, influence other members of the network so that A and B can ultimately influence each other inside it. If A is simply connected to B, C, and D (which are interconnected), receiving information from them, but A cannot send information to B, C, or D, then A is not part of the BCD strongly connected component and therefore cannot be part of its identity. Note that there is a significant difference between A being part of an interactive identity and A being identified—or even identifying—with it. If A is an actor that simply happens to follow a given network activity and is influenced by it but cannot influence it back (directly or indirectly), A might symbolically identify itself with that network but it would not be part of its interactive identity. Conversely, A may be part of such an interactive identity without knowing it, even opposing identification with and despising it (i.e., if A influences B, C, or D, and *vice versa*, no matter how little A identifies with it symbolically, it can be part of its operational identity, even if antagonistically integrated).

In Figure 3, we can distinguish two identity cores, one for each community. Nodes n0, n9, n16, n18, n34, and n42 are interconnected so that they all influence each other—they form the strongly connected component of the red community A, whereas nodes n1, n4, n7, n23, n26, n40, n41, and n47 form the core of the blue community B.

#### Identity Audiences and Sources

##### The nodes of the community of a given identity core can become an audience of the identity if they receive information from the core or a source if the core receives information from them

As mentioned before, if node A does not feedback interactively with strongly connected nodes B, C, and D, it does not constitute that identity, but it might be part of its community. There are two major forms in which this can happen: node A can follow the identity core, consume its information, and amplify its reach or it can be a source of information for the core identity, without itself being affected by the activity of the collective identity. The nodes of the first group make, what we call, the “audience” of the collective identity and those of the second we call the “sources” (which we will use as a short for “source of information” with no intention to denote the origin or essence) of the identity core. Note that audience nodes are not all those that receive information from the core, but only those that belong to the community. In other words, if A receives information not only from an identity core but also from other agents or other identities or communities, node A will not be considered an audience. So, for example, in Figure 3, n44 receives information from n26 (which is part of the core of identity B), yet it is not an audience because it also receives information from nodes 5 and 25. Node n37 only receives information from identity core A and thus becomes an audience^9^. Note that n30 is also classified as an audience: it receives influences from node n0 at the core and also from the resource n3. Nodes are sources of a core identity if they belong to the community and if they primarily feed the core identity more than they do other nodes or groups or nodes in the network. In Figure 3, nodes n35 and n21 are sources of identity core B; the case of n43 is interesting because it is a resource of core B and it feeds directly into a resource of community A (n24) and environmental nodes n5 and n36.

Note that sources and audiences can have depth. A node n can be a source of a source of the core identity of community A or audience of an audience. There exists as well a third type of nodes in the community that are neither source nor audience because there is no direct information flow to or from the core identity, but they exchange information with audience or source nodes. These cases occur when node X can be a source of an audience of node Y of core node Z, but neither source or audience of the core and conversely node X can be the audience of node Y that is a source of core Z but not an audience or source of Z and also in all depths of previous cases. These cases are not displayed in Figure 3 but will appear in the empirical cases below.

#### Identity Cohesion and Internal Structure

Strong connectedness is the most basic or relaxed condition for an identity core. It is possible to deepen into the strength of the collective identity by means of other network theoretical properties. A central one is the notion of *k-connectedness*, which has been matched with cohesiveness as a key feature of social groups and networks (White and Harary, 2001; Moody and White, 2003). Moody and White (2003) define the relational (as opposed to the ideational) togetherness or *structural cohesion* of a group as the extent to which “the social relations of its members hold it together” (p. 106) and determine that “a group’s structural cohesion is equal to the minimum number of actors who, if removed from the group, would disconnect the group” (p. 109). This definition corresponds to the network theoretical concept of *k-connectedness*, and it further allows splitting of the identity core into nested cohesive blocks. In turn, *embeddedness* into a collective identity is the individual counterpart of structural cohesion: the deeper a node is situated in nested cohesive blocks, the higher its embeddedness. Following their work, we consider that the cohesion of a collective identity core can be measured by its *k-connectedness*, that is, by the number of agents that needs to be removed to disconnect the core^10^.

Note that this definition makes identity cores that depend on one or two strong leaders very weak in terms of cohesiveness. In this sense, k-connectedness can also be considered as an indicator of the degree of collectiveness of the identity core. A core with a single leader that holds the group together is much less collective than that of a highly interconnected core where multiple paths exist between any two nodes to inform and affect each other and no single node holds the key to maintain the whole.

## Application to Three Case Studies: 15M Indignados Movement, Spanish 2019 General Elections, and General Strike for Catalan Independence

We now apply this characterization to two case studies of (techno)political identity formation on Twitter and one on Facebook. The idea is to show practical applications of the theoretical construct in different spaces, scopes, and structures. In particular, we study three types of collective identities: those associated with political parties, those tied to different poles of nationalism-loaded debates, and those of social movements.

The three case studies display limitations due to data collection constraints and sampling methods. Despite these limitations, our approach is able to depict consistent collective identities and their internal structure, yet the limitations on data sampling methods should not be confused with definitional procedures. In particular, defining collective identities within a scope was not intentionally translated into any specific procedure to collect data. Defining a scope is not always trivial. Moreover, even if a scope is well defined, technical problems might preclude its application. For example, if the scope is well specified by means of a complete set of terms, the resulting query to social network platforms often finds data processing limits or, even if the data is accessed, its processing is too costly. On the other hand, if the scope is well defined by some natural language processing algorithm, the whole unlimited conversation data would be required to apply the algorithm. In our case, we had to make data sampling decisions or work with existing datasets that did not perfectly match our notion of scope (within which collective identities are to be found)^11^.

### Spanish General Elections 2019

We collected data through the Twitter Search Application Programming Interface (hereafter referred to as API^12^). The possible public communicative interactions that define the interaction space of this platform are creation–emission (tweet), access (read), response (reply), and re-emission (retweet) of short digital messages in a message exchange network. The API makes it possible to retrieve tweets containing words of any given set. Thus, the set of keywords used to retrieve the dataset defines, in this case, the interaction scope under analysis. We use as keywords the twitter handle and names of candidates and political parties participating in the Spanish general elections in April 2019. In particular, our dataset is composed of tweets (and retweets) emitted during the 3 weeks spanned by the official electoral campaign (8th to the 27th of April 2019). Thus, the data collection method is both node- and topic-centered. Individual agents are Twitter handles, being them persons, collective organizations, or bots. While different types of interactions are possible in Twitter, we restrict the analysis to retweets.

We represent the interaction networks with twitter handles as nodes and directed links from node A to node B if A retweeted B. We associate a weight to the link directly proportional to the number of retweets done by A to B.

In order to identify a first level of systemic integration, we isolate the *giant weakly connected component* (the ecological connectedness). As a second step, we filter the network according to the level of significance of the interactions. To do that, we apply a disparity filter and retain only those links which beat the (local) threshold. Since our links (interactions) are directed, we can consider the significance of a link from the point of view of the sender or from that of the receiver. When filtering, we always take the highest of incoming or outgoing links from a node. Following the nomenclature in network science, we call the filtered network the *backbone* network.

The giant weakly connected component of the backbone is composed of 133,734 nodes/twitter handles. Those are the individual agents of the system under analysis. We now apply a community detection algorithm to the backbone network. The first 10 communities by size represent more than 80% of the system, and we restrict our analysis to them. As we can see in Figure 4, each of the main communities can be identified with a political party or, in one case, with a group of political parties that share a common goal in scale and in relation to all other parties (this is the case of Catalan independentist parties).

We calculate the strongly connected components to isolate the core identity of each community. To assign a specific political party to a community, we look at the party or candidate profile included in its strongly connected component. Then, we identify the audience and the sources with respect to that strongly connected component or core identity. In some specific cases, we also consider in some detail other core identities (we call “secondary”) that exist within the same community. We consider that a community does not contain or constitute an identity if the strongly connected component within it has less than three nodes.

The resulting political identities basically correspond to the most important political parties in Spain and a further Catalan independentist identity composed by different Catalan political parties. We have also identified a community of news media as a shared source for different political identities.

Vox (extreme right) and Podemos (left), both relatively new political parties in Spain, are the first and second communities by size, representing, respectively, 15 and 11% of the whole network. However, their identity cores are small compared with that of traditional parties (PSOE and PP), both in terms of relative and absolute size. In the case of Podemos, audience represents 72% of non-identity core actors, while in the case of Vox, it represents 49%, and the largest part of actors (51%) is neither source nor audience. This is because there is no directed path from the identity core to them or *vice versa*. It is important to note that, in both cases, secondary core identities are larger than the ones that include official actors (parties and party leaders).

The largest political core identity, in relative and in absolute size, is that of PSOE, the party who won the elections (representing 4% of its community and 0.3% of the whole network). Also, it is the most cohesive, its maximal k-core being equal to 15, while the PP identity core has a maximal k-core equal to 10, Vox has equal to 7, and Podemos has equal to 4 (see Table 1 for a comparative summary). The identity is composed by party candidates, party official accounts, and also other agents (mostly “ordinary” supporters and non-public figures of the political party). During the electoral campaign, most of the supporters adopt a banner of the party in their profile picture, along with a campaign hashtag in the bio, as a sign of political identification. The large majority of ordinary supporters included in the identity core adopted these signs.

**TABLE 1:** Main network properties of the collective identities corresponding to the most important political parties in Spain during the 2019 election campaign on Twitter.

**Party**	**Community (% of the total)**	**Identity core (% of the community)**	***k*-core**
Vox	15%	0.1%	7
Podemos	11%	0.2%	4
Psoe	7%	4%	15
PP	5%	3%	10

*We quantify the size of communities within the whole election campaign network, the relative size of their identity-cores and their cohesiveness (measured as k-core).*

The third community by size is ascribed to the Catalan independentist political identity, which was composed of different political parties. Due to the relatively small size of Catalunya’s region in the context of all Spain, they all appear bundled on a single identity. However, the identity core is formed by just four nodes, all connected to all, around the exiled former Catalan president, Carles Puigdemont (KRLS), that is in the core. Finally, the sixth community can be ascribed to the political party Ciudadanos, but it displays no identity core.

Figure 5 focuses on a specific political identity (that of Podemos) to show its internal structure. Its core identity is composed 36 nodes, including official accounts of public figures and members of the head of the political organization. The most embedded account includes the candidate for president (@Pablo_Iglesias_), the secretary of the organization (@pnique), the official account of the political party (@ahorapodemos), and speech persons (@ionebelarra and @Irene_Montero_). Some prominent figures of the information source of the identity are Joan Mena and Monedero. Monedero was a former member of the core organization of Podemos until his resignation from the Podemos political steering committee. Joan Mena is a member of the Catalan political party (En Comú Podem) that forms part of the bigger Unidas Podemos coalition. The analysis could be extended in more detail, but we have shown that the distinction between source, core, and audience is valid and consistent between and within political identities as depicted through some technopolitical interactions in Twitter.

### Two Identities in Confrontation: General Strike Against Trial to Catalan Government Members

For this case study, data were collected during the general strike in Catalunya on the 21st of February 2019, with a set of related hashtag as keywords for querying the API. The strike was called by a Catalan independentist union against the judicial process of independentist activists and politicians. With this dataset, we expected to find, making use of our definition, two types of collective identities in opposition, which would stand for the independentist and anti-independentist sides qualitatively recognized in the confrontation.

As we have mentioned above, most community detection algorithms have so-called *resolution parameters* that control the number of communities that result from the application of community partitioning methods. Variations in the resolution parameter result in different partitions of the networks with different numbers of communities per partition; however, for a partition to be accepted as significant, a quality parameter has to be checked. In this case study, we are using the Louvain algorithm with resolution parameter, and we accept partitions with a modularity value above 0.4 and with less than 200 communities^13^.

We expect to have two main identities that eventually may have some internal structure or sub-identity. To check for this hypothesis, we calculate communities by varying the resolution parameter between 1 and 5, 1 being the *default value*. We use this case study to show how, despite the potential ambiguity of the way in which the community partition algorithm’s threshold might “arbitrarily” split a given interaction network and thus the underlying identities, community (or modular) partitions are often stable for different parametric configurations and reliable to characterize the relevant identities within the network.

**FIGURE 6:**
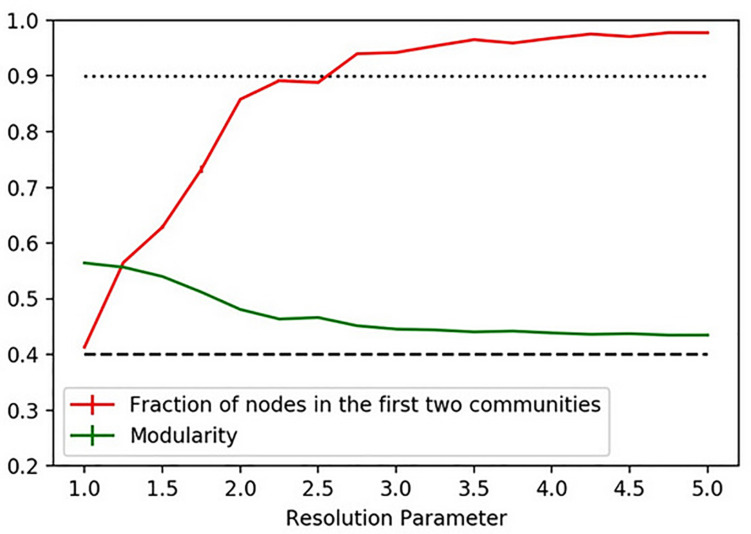
For each value of the resolution parameter, we calculate 10 partitions. The mean fraction of nodes in the first two communities and the mean modularity are respectively in red and green. The error bars representing standard deviation are almost invisible because they are smaller than the line’s thickness.

From Figure 6, we can see that above the value 2.5 of the resolution parameter, more than 90% of the nodes are in the two main communities, while the modularity index remains above 0.4 (which is considered as sufficiently high). For greater values of the parameter, we appreciate a moderate increase of the percentage of nodes in the first two communities while the modularity is almost stable above 0.4.

From this, we conclude that the system presents a robust bipartition. However, at the same time, we also note that, for small values of the resolution parameter, we have the modularity approaching 0.6 and the nodes dispersed in more than two communities. We can interpret this by recognizing that the system presents different structures at different scales of granular analysis (like that of tissues, cells, and organelles under a microscope).

**FIGURE 7:**
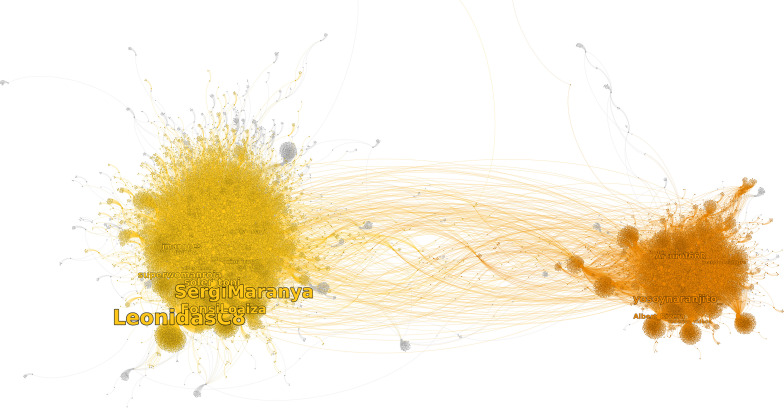
Nodes are twitter handles, links represent retweets, and the direction of the link is indicated by the curvation, with the direction being aligned in a clockwise direction. The colors represent communities.

We now look for identity cores, i.e., strongly connected components, at the scale at which the system is bipartite. For this, we take a partition with the resolution parameter set to 3. The first community represents 62% of the entire network, and the second represents 33% (as shown in Figure 7). The largest community is associated with the movement calling for the liberation of the arrested Catalan politicians and activists, while the other one is associated with Spanish nationalists or constitutionalists (defending the constitutional unity of Spain).

Inside both communities, we found two strongly connected components representing, respectively, 1.3 and 1.5% of their communities. The two other strongly connected components represent less than 1%; thus, we do not analyze the system with respect to them. Audiences represent, respectively, 52 and 48% of the communities, and sources represent 1.48 and 1.62%, respectively. Thus, the two communities, even if they differ in size, are quite similar in roles and compositions.

In both identity cores, the most central node is a Twitter activist, LeonidasC8 and yosoynaranjito_, respectively. Also, in the first 10 positions, we have activists, political organization, and civil society organizations on both sides. However, they differ in the composition of sources, being mostly news media for the independentist community while politicians from different political positions for the constitutionalist community; in particular, we found Albert Rivera, from the liberal right party Ciudadanos, in the first position by centrality and José Zaragoza, from the socialist party, in the fifth position.

When partitioning the network with a lower resolution, the constitutionalist community remains more or less identical, with two small additional communities appearing, both centered on the previously mentioned politicians and the supporters of their respective political parties or social-democratic and liberal-conservative Spanish constitutionalists. On the other hand, the other Catalanist side breaks in three communities of comparable size. One was centered around the Twitter activist that leads the identity core in the bipartite phase, one was formed by the political party CUP and its audience, which were in the former identity core, and a last one that was centered on the remaining part of the former identity core. Interestingly enough, a new community appears with an identity core, and it can be related to Twitter activists proximal to Podemos.

### Multitudinous Identity: 15M Indignados

We close this application section with a different interaction space, scope, and type of underlying collective identity. Here, we study the network of Facebook pages of actors related to the 15M indignados Spanish movement [data and network characterization were taken from Monterde et al. (2015)].

The interaction space is Facebook, while actors are pages of collectives or initiatives. The methodology followed for sampling the network was node-centered. Based on situated knowledge, the authors chose a set of initial pages that they used as seed for a snowball sampling algorithm. Starting from these seed pages, they added as new nodes those pages liked by the original ones. This step was further repeated. If page A liked page B, a connection A → B is established [see Monterde et al. (2015), for more details]. The interaction scope is thus here defined not by the content of interactions but extracted from a sampling origin. In order to provide some comparative contrast, the seed also included the official fanpages of the biggest Spanish labor unions, again with depth 2 of their like network.

The sampling method is different from the one used in the previous cases of study. First of all, like relations between pages is a binary relation, it is present or not and cannot be associated with a weight. Because of that, there is no possibility to filter relations according to their significance level. As we will see, this results in few communities with more broad identity cores and tiny audiences. The sampling method also affects the composition of the identity core since seed pages have a higher probability to enter the core.

**FIGURE 8:**
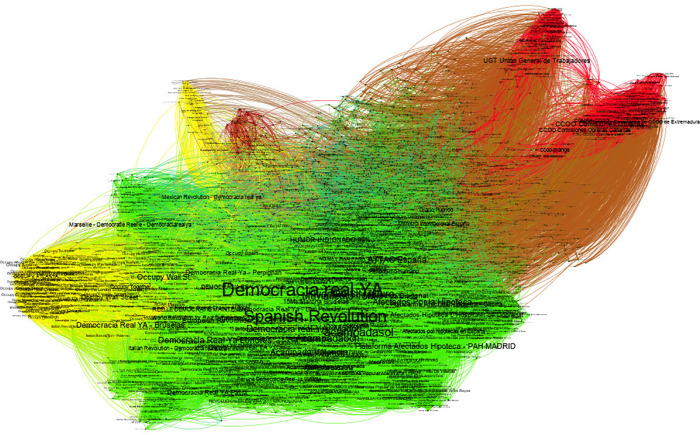
Nodes are Facebook pages, links represent likes, and the direction of the link is indicated by the curvature of the connection (in a clockwise direction). The colors represent different identities: green for the Spanish Indignados 15M, yellow for Occupy movement in the United States, and red for Spanish Unions UGT and CCOO (selected to contrast with the 15M collective identity).

**FIGURE 9:**
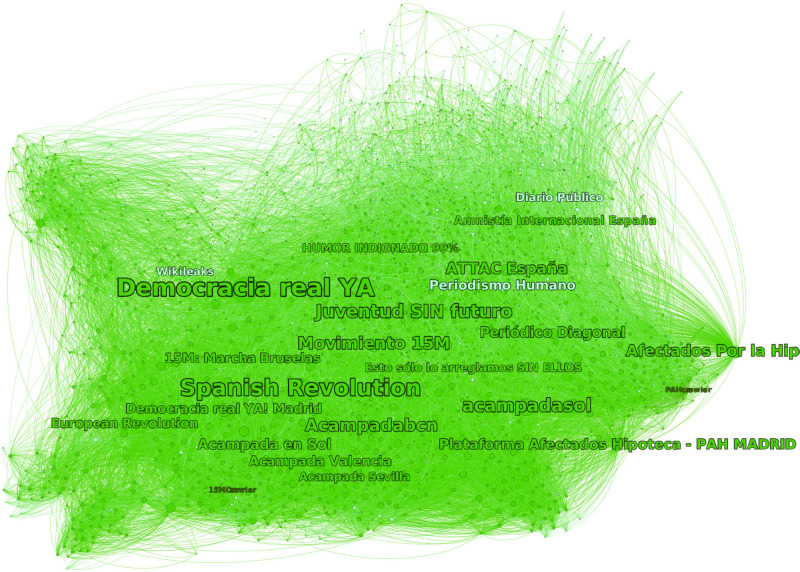
A closer look at the 15M community and its identity core and sources: Wikileaks, Periodismo Humano (Human Journalism, a Spanish web-based alternative news media), and Diario Público (newspaper).

The largest community represents 54% of the network and is organized around the most central pages that are pages of movement organizations (displayed in green on Figure 8). The identity core of this community includes 60% of the pages. The source includes 98% of the pages not in the core (see Figure 9). Even if some effect of the sampling method and of the interaction space may be present, this is a strong indicator of the reciprocity attitude of this identity since this is not the case of others. This is also reflected in the high cohesiveness of the identity core; the maximal core number is 40, and 30% of the nodes are in the maximal *k*-core.

The third largest community is formed by Occupy movement-related pages and pages of indignados movement outside Spain (displayed in yellow color in Figure 8). It represents 8% of the network. The identity core represents 70% of the community. The source represents 78% of the rest of the community, while the audience there represents 18%. Here the organization of the identity and its relations is similar to the main community. The nodes belonging to this community arose spontaneously on the dataset as tied to the 15M seed snowball sampling.

The second largest community is the result of an explicit seed, organized around the two big Spanish unions and representing 33% of the network (colored in red in Figure 8). It was introduced to work as a contrast and more traditionally organized environmental identity for 15M. The identity core represents 36% of the community, while the source represents 99% of the rest of the community (excluding the core), the audience being less than 1%. Here, we observe a strong directionality on the relation between the identity core and others, with a tendency of the core to engage in relations with others, but not the other way around. Given the dimension of the core, a hypothesis could be that sources are those that choose not to be in the identity core by not liking back pages in the core. It is also important to acknowledge here the limitation of the like connections in Facebook for the application of our definition that would rather demand a more interactive information flow.

## Discussion: Collective Identities Through (Technopolitical) Interaction Networks

To our knowledge, we have provided the first fully operational interaction-centered definition of collective identity and its internal structure. It is certainly not complete, but once an operational definition is made explicit, albeit partial or incomplete, the benefit is that conceptual, mathematical, or algorithmic improvements can be made on specific unambiguous grounds and, similarly, assumptions and consequences can also be made explicit, discussed, and modified. In response to the criticisms to the use of the concept of identity, because of its slipperiness (Brubaker and Cooper, 2000), we believe that our work has first served to systematize and clarify the different approaches, values, and contemporary opportunities to study it [Section “Mapping (Collective) Identity: A Brief and Broad Approximation”], then to operationalize it (Section “Proposal: Collective Identities as Strongly Connected Cores Within Communities and Environments in Digital Interaction Networks”), and finally to apply it to various case studies (Section “Application to Three Case Studies: 15M Indignados Movement, Spanish 2019 General Elections, and General Strike for Catalan Independence”). In this section, we discuss some conceptual or theoretical progress that can be made departing from our proposed definition of collective identity, and we put it in connection with the wider theoretical landscape depicted in Section “Mapping (Collective) Identity: A Brief and Broad Approximation.” We also suggest some future lines of methodological, experimental, and conceptual improvements that can be used to expand the present framework.

### A Working Operational Definition for Different Types of Collective Identities in Technopolitical Interaction Networks

In our analysis, we studied three different cases. One of them is a case of party technopolitics (2019 April general elections in Spain) and the other two of contentious technopolitics (Catalan strike and 15M). Elections are a prime example of the competitive and pluralistic moment of party politics (Bourdieu, 1981; Dahl, 1982), where different organizations launch their messages and try to mobilize their constituencies (or gain new ones) around a shared set of topics. Differently, the Catalan strike displays a bi-polar and antagonistic moment (Laclau and Mouffe, 1985) of contentious politics, when two groups opposed around a matter of dispute directly confront each other and split in two the political space. Finally, in the 15M case, we can see the self-constitution of a multitude through networked interactions (Monterde et al., 2015).

In this paper, we wanted to focus on the way in which each of the collectives involved in these variegated forms of politics can, despite their differences, be subsumed under a unified operational definition of *collective identity*. We have successfully shown that to be possible. Regarding the three major families of approaches to the notion of collective identity (essentialist, ideational, and interactive or relational), we have shown how, at least within the type of digital interactions studied and filtered by scopes, it is possible to precisely characterize collective identities in terms of the topological analysis of interaction networks without references to the shared properties of the constituents nor any specific understanding of their psychological representational identification and without any explicit analysis of the content of their interactions.

Our approach has put the emphasis in the commonalities of the ways that they do so. We have shown a shared pattern of current party and contentious technopolitics: the internal anatomy of interactional processes of identity formation through networked communication. We have shown how the type of platform may shape some specifics but that the key elements of collective identities (communicative interactions, cores, and audiences) may be relatively independent across different platforms and for different forms of politics (electoral, antagonist, and multitudinous).

However, our framework can be shown to do more than characterizing different types of interaction clusters under the unique and consistent operational concept of collective identity; it can also point to important differences. We can see that, in parties, the core identities are rather small (from 1 to 4%, depending on the party), while in the movement case, they are huge (up to 70% of a given community). This suggests a feature not underlined in our previous paper (Monterde et al., 2015): the identities in movements such as 15M tend to reduce the “leadership” (core) vs. “audience” divide, incorporating the latter into the former and transforming the typical asymmetric shape of communication in political representation into one closer to the ideal symmetrical shape of political participation. The 15M core identity indeed looks more multitudinary than the elitist core identity of parties. This may have another implication: the self-communication around parties looks closer to the traditional mass communication (one-to-many) model, even if it may count as *mass self-communication*. Meanwhile, the 15M seems to take a form closer to what we earlier defined as *multitudinous self-communication* (a fully developed many-to-many model). However, there are a number of caveats to notice around this result. We touch upon them in the following section.

### The Complexity of Technopolitical Inter-Identities: Multidimensional, Multilayered, and Multiscale

The question arises as to whether interaction networks alone *constitute* collective identities or if they are “only” an increasingly measurable aspect of sociality that becomes useful to characterize those identities. In other words—are we suggesting that our proposal is an epistemic tool or a description of what collective identities really are? In a sense, both statements are partially correct. The interactive and rather structural(ist) conception of collective identity that we have proposed is not reductionist. We believe that identities are *multidimensional* and so should be the approaches to them. Forms of social identity cannot be explained away by interaction structures alone; the meaning of such interactions (the ideational dimension of such interaction) is crucial to the formation, maintenance, and transformation of the interaction structures themselves, the collective identities they give rise to, and the way in which collective and personal identities continuously feedback to each other. Moreover, the very identification of interactions (what is an interaction) and their selection is not without a certain semantic, ideational, or interpretive load (embodied on a selection of samples, scope filters, etc.).

Interaction structures are not simply a passive fossil or trace of the symbolic exchange and the associated identification processes. The interaction structures (and the platform infrastructures underlying them in the digital domain) also shape the ways in which agents build and re-negotiate their cultural meanings, generate collective claims, create new symbols, and preclude or amplify psychological and social effects [f.i.: the degree of embeddedness, affection, and salience of the social identity of individuals (Ashforth and Rogers, 2011)]. Without interaction, there is no organization, and without organization, any “essential” identity remains a passive collective trait, while ideational or representational identity (f.i.: self-ascribed identity) remains disembodied and inert. Thus, although recognizably incomplete, a proper interaction-centered operational characterization of collective identities is not only possible today (as we have shown) but also necessary to properly understand social identities in their full complexity. This complexity requires, in turn, more detailed examination and acknowledgment of its various facets.

Due to the *multilayer* or multi-space configuration of the underlying collective identities, what we have termed as identity audiences should not be dismissed as causally irrelevant or epiphenomenal in the conformation and the evolution of a socio-political identity. First of all, audiences are always potential cores and might also display as audiences in a given time span or a specific interaction space or scope while being part of the core at a larger timescale or a different interaction space. Also, particularly in politics and more so in representative democracies, audiences play a significant role outside any public-sphere communication domain: voting, and yet this is certainly not the only layer that matters to political collective identity formation. Although certainly informative, the study of interaction dynamics in an isolated interaction space is but an indication of deeper and more complex phenomena that are built across different layers (Kivelä, 2014; Cozzo et al., 2018). From a theoretical perspective, many studies show that both structural organization (Cozzo et al., 2015) and dynamical outcomes (Cozzo et al., 2013) look different when multiple layers are taken into account.

Collective identities are also *multiscale*. We have focused on a single scale of identity formation, but social and collective identities often appear nested (Ashforth and Rogers, 2011; Ashforth and Johnson, 2014). So, for instance, the electoral collective identities were identified at the scale of political parties (and the stronger and clearer network divisions appear at that scale), but left–right identities can also be depicted as merging different collective identities (those of political parties) into the same super-identity. This is partly inevitable and, instead of a methodological flow, it describes a property of social systems where multiple scales of identity or nested identities coexist. They go from the individual up to the whole of society, from the micro to the macro. Interestingly, the resolution parameter of community detection algorithms is crucial into freezing a specific scale, and our proposal to operationalize collective identities can accommodate and measure the capacity of social systems to organize into nested collective identities and communities.

In the broader picture of how identities (from personal identity to role and social identity to collective identity) are nested at different scales of interaction (as depicted in Figure 1 at the beginning of this paper), our contribution falls short to unpack the full complexity of human identity formation. However, by clearly depicting spaces and scopes, community boundaries, and network identity structures, it is now possible to address specific questions as to how the different levels of interaction might relate in the construction of human identities (e.g., how is role identity conditioned by the specific embedded positions within the collective identity network?).

### Limitations of the Current Approach and Possible Improvements

There are a number of limitations and potential improvements to the cases and the methods presented in this paper. Some are of a technical and methodological kind. In particular, more detailed studies are needed to detach the effect of the sampling methods on the observed organization of the interaction network. In this direction, the development of a sampling method that is theoretically and statistically well-grounded to target collective identities is an urgent task.

We suggested studying collective identity as a *network that is both the result and the source of recurrent, cohesive, and coordinated communicative interactions*, but we focused exclusively on the interaction structure, the topology of relationships emerging from interaction networks, averaged out or mapped into a unified structure, and we have focused on studying a short period (“snapshot”) of interaction structures, that is, without considering its evolution over time. Despite the difficulty of gathering the data, a deeper study of collective identities in digital networks should include the long-term processes of evolution and structural change: the formation, split, expansion, extinction, etc., of collective identities. A further development of the notion of collective identity could and should also be enriched with the study of the dynamics of coordination and integration of interactions. The timing between the interactions, not only their structure, is, in this sense, informative. Temporal correlations, synchronizations, delays, etc. play a very important role in characterizing the degree and the quality of the coordination between individual agents (Aguilera, 2018).

Further improvements would demand that we apply our definition to more cases (more typologies of social and political collective identities) and also to a wider set of interaction spaces (other platforms, forums, social networks, mailing lists, etc.). Moreover, we have only studied collective identities within a single space while acknowledging that they develop across different spaces. So, studying the same collective identity across different communication layers and platforms (Twitter, Facebook, Whatsapp, etc.) remains an avenue for future research.

It also rests to be seen whether the present approach to collective identities is applicable beyond digitally mediated interactions and useful to other sources of interaction data (conversations, encounters, meetings, etc.). Of particular interest to us is the comparison between digitally mediated identities and the more traditional ones like those potentially emerging from traditional mass media, face-to-face meetings, or other means of communication.

Beyond the “interactionist” analysis in this paper, current computational methods also make it possible to take a more “ideational” approach [closer to the relationist tradition mentioned in Section “Mapping (Collective) Identity: A Brief and Broad Approximation”] by looking at the content of the symbolic exchanges to the formation and the characterization of collective identities by studying how collective claims evolve in parallel with the network structure of collective identities. Interactions can also be valued (in positive or negative terms, in their strength, etc.) by introducing yet richer values to network edges on the basis of automated content analysis (like sentiment analysis), and it is also possible to study identification together with interactional identity attending to the content of discourse frames [see Gallagher et al. (2018), for an example]. Such improvements go in the direction of extending the analysis from the syntactic (the interactional) into the semantic (the ideational) and potentially the pragmatics of technopolitics (Calleja-López, 2017).

One key to reach a more general theory of collective identities is the distinction between different *types* of identities and their relation with different models of communication. A specific limitation of the present paper lies in the fact that both the platform and the type of interaction (retweeting vs. following) are different in the 2019 elections and the 15M cases. Exploring such differences, using datasets from a single type of interaction and platform and adding the mentioned ideational (or semantic) aspects, is crucial to explore the possible types of identities and their relations with different forms of self-communication.

### Analysis and Synthesis of Collective Identities in Technopolitical Interaction Platforms

We believe that digital platforms both mediate and simplify the ways in which social identities emerge. The platforms’ mediation has a clear constructivist potential: platform affordances and performances seem to shape social phenomena (Bucher and Helmond, 2017). In this sense, technopolitical inter-identities partially express the technical conditions underlying them. One key effect is their simplification: people can only perform a set of defined tasks. Combined with the legibility afforded by the platform, this makes possible a precise mapping of formal human and non-human behavior. We believe that, today, a systemic and network theoretic approach to collective identity brings the notion closer to the operationalization that some authors demand (Opp, 2009).

One of the most relevant questions from a technopolitical point of view is the manner in which different interaction interfaces and mechanisms might constraint and enable the dynamic emergence of collective identities—for a distinction between mechanics, dynamics, and esthetic in computer game theory, see Hunicke et al. (2004). Social network engineers and user interface designers determine a set of mechanisms (information fields, possible digital actions, user relations, channels of information flow, etc.) and interfaces (position, color and size of buttons, counters, fields, types of feedback, etc.) that deeply influence the kind of dynamics that emerge on the platform. Whereas Twitter like interaction spaces, made of short messages, quick interactions (retweets and response), and a continuous timeline, probably favor large networks and the fast formation of collective, technopolitical inter-identities, it is highly probable that they come at a price of low deliberative quality, lack of long-term cohesion, and fast confrontational dynamics.

Questions arise as to what kinds of interaction mechanics produce or facilitate the emergence of more (or less) cohesive, open, adaptive, sustainable, and diverse collective identities and how could a change in interaction mechanisms induce a resolution or lower the confrontation between two identities or break false consensus and visualize underlying social confrontations that are otherwise hidden. Our approach makes it possible to address these questions and to better design technopolitical networks with the goal of enriching the diversity and the complexity of social identities. Simulation models of network dynamics and multi-agent systems could provide valuable insights in this direction.

### Why an Operational Approach to Technopolitical Inter-Identities Matters

Already in the 1970s, it was suggested that “the presently existing, largely categorical descriptions of social structure have no solid theoretical grounding; furthermore, network concepts may provide the only way to construct a theory of social structure” (White et al., 1976, p. 732). Although we only partially agree with this position, the transition toward digital social networks has strengthened some of the possibilities (and, in some senses, revealed the limits) afforded by network approaches. This takes place, particularly, in two respects. Firstly, digital networks and interactions can be mapped in detail. Secondly, the types of interactions afforded by digital platforms are limited, thereby simplifying and clarifying the structures and the dynamics of social relationships. There are various reasons for why our approach to collective identity matters today: (1) digital infrastructures make it possible to connect, disconnect, and reconnect, i.e., to redefine the interaction structure of communities, faster and more distributed than ever before; (2) the structure of such interaction networks is increasingly more available to study and manipulate; thus, it is likely that interaction structures become more central to the emergence of collective identities not because of any ontological priority status but because they might become a more direct object of action, representation, intervention, and explicit design; (3) the increasing prevalence of digital platforms that mediate social interactions puts pressure on the way in which such platforms are designed and regulated, yet regulating in terms of content (within the boundaries of basic human rights) is problematic. Efforts should be made to intervene primarily on the interaction mechanics that afford the emergence of social structures (from contagion to identity formation). To provide interactionist operational tools to measure and characterize collective identities is increasingly relevant if we are to defend the diversity of identities and deliberative quality. This is particularly relevant now that public attempts are becoming increasingly successful in creating digital platforms for distributed, deliberative, and participatory democracy (Barandiaran et al., 2017, 2020). Interface and interaction mechanics design is crucial for the emergent dynamics of decision making. We need a theory of the kind of network structure that is more democratic, making it possible for identities to emerge, express conflict, solve it, and increase their autonomous agency; and (4) it is more and more common for social and political movements to represent their own identity as a network of interactions. Network diagrams are not only epistemic tools but also ideational tools themselves, and providing a precise algorithmic procedure to generate such representations is an important part of identity formation processes.

## Recapitulation and Conclusion

In the era of artificial intelligence and algorithmic governance, through the combination of corporate social networks, big data analytics, and political cyberwar, the impact of digital networks on political life and social identity formation is becoming increasingly problematic. Interaction-centered approaches to identity formation not only make it possible to study such phenomena but they also allow to define protective and social autonomy-enhancing measures against the way in which corporate and institutional powers can asymmetrically intervene on the way we collectively define who we are. Operationalizing and quantifying the emergence of collective identities in digital interaction networks is a fundamental quest in this direction. To the extent that the increasing platformization of society extends and plays ever bigger roles in society with the increasing social penetration of digital platforms, the approach to identity that we take in this paper will probably gain relevance in time. Our analysis will gain applicability with the growing platformization of the social. Beyond its epistemic value, we believe that our approach is also useful for grounding critical evaluations and alternative models of design.

In this article, we have characterized a conception of collective identity that takes advantage of interactionist and neo-structuralist approaches through social network analysis. Inspired by the way in which the concept of identity is cast in complex system approaches to life and mind, we have provided an operational definition of collective identity and have shown how it successfully applies to different cases and domains. The proposed framework can be improved methodologically by including a dynamical analysis of interactions. It could also be complemented with computational methods that tackle ideational aspects of collective interactions and would certainly benefit from further experiments with richer and temporarily extended datasets.

## Data Availability Statement

The datasets generated and analyzed for this study can be found in the IAS-Research github repository https://github.com/IAS-Research/defining-collective-identities-datasets.

## Author Contributions

XB has led the research and content of the manuscript and its general conception, developed the operational definition and toy model of collective identity (section “Proposal: Collective Identities as Strongly Connected Cores Within Communities and Environments in Digital Interaction Networks”), and coordinated theoretical and experimental integration and the overall writing of the manuscript [writing sections “Introduction” and “Proposal: Collective Identities as Strongly Connected Cores Within Communities and Environments in Digital Interaction Networks” and parts of sections “Mapping (Collective) Identity: A Brief and Broad Approximation” and “Discussion: Collective Identities Through (Technopolitical) Interaction Networks”]. EC has provided technical guidance on the development of the proposal (section “Proposal: Collective Identities as Strongly Connected Cores Within Communities and Environments in Digital Interaction Networks”), carried out the application of the framework to case studies together with data collection, analysis, and representations, and handled the overall revision of the technical and the conceptual issues throughout the manuscript and writing of section “Application to Three Case Studies: 15 m Indignados Movement, Spanish 2019 General Elections, and General Strike for Catalan Independence.” AC-L has contributed to the framing and theoretical aspects of the manuscript, detailed discussion of definitional aspects, theoretical implications and consequences of technical decisions, writing sections “Mapping (Collective) Identity: A Brief and Broad Approximation” and “Discussion: Collective Identities Through (Technopolitical) Interaction Networks,” and reviewing the whole manuscript. All authors contributed to the article and approved the submitted version.

## Conflict of Interest

The authors declare that the research was conducted in the absence of any commercial or financial relationships that could be construed as a potential conflict of interest.

## Footnotes

1 The 15M/Indignados was a social movement in Spain, specially strong in the period 2011–2013, which made an intensive and creative use of social media (Hughes, 2011; Toret et al., 2015) and opened a political cycle that involved the creation of new parties such as Podemos and new forms of municipalism (Feenstra et al., 2017).

2 The brief review in this paragraph is based on Gleason (1983), Brubaker and Cooper (2000).

3 In a more complex manner, since the work of Marx and Engels, class could be understood as a progressive construction that goes from structural conditions under capitalism (that define a class *in itself*), through relations between actors (the class *by itself*) up to the rise of collective class consciousness (the class *for itself*).

4 For Tilly, unlike interactions, transactions constitute the actors involved in them: in transactions, actions run through the actors and not merely *between* them.

5 It might be important to clarify, at this point, the type of interactions that we are seeking for and what is required to be analyzed from such interactions. We can illustrate the nature of such interactions from those we are about to study: retweets in Twitter short messaging networks. For those who are unfamiliar with Twitter, a retweet might be thought of as equivalent to forwarding an email to your contact list or repeating an idea or argument you might have heard somewhere to all your family, friends, and workmates. What is important to note is that we do not study the content of the Tweet or idea being spread but the “mere” fact that person A retweeted to C and D what B said. (Thanks to reviewer 1 to point out the need to clarify this point).

6 The scope is very often specified, filtered, or selected during the data collection process by some sort of keyword filtering (hashtag, scope-specific terms, etc.) or network sampling algorithm (Carrington et al., 2005). Broadly speaking, the data collection methods for a social network study can be divided in two classes: node-centered and topic-centered methods. In node-centered methods, one or more nodes are used as seeds and the relation of these nodes with others are exploited to explore the networks, while in topic-centered methods, the content created by social actors is used to discover relevant nodes and their relations. A typical example of node-centered methods is the various types of snowball sampling. In this type of sampling, one starts with a set of central nodes and then explores its alters (nodes connected to members of this set) and includes or discards them (according to certain criteria, like strength of the connection or certain attributes of the alter node) to be included in the set and proceeds recursively with other members of the setup to a certain depth. This kind of algorithm is often used to study “hard-to-reach” populations/identities (Baltar and Brunet, 2012). In this method, the selection of the seeds and of the inclusion criteria delimits the scope. On the other hand, in topic-centered methods, a set of keywords is used to filter the content produced by actors, thus including in the sampling all actors that use the keywords and the relations among them. In that case, the keywords set is the delimiting proxy for the scope (Schmidt et al., 2018).

7 In particular, we will adopt what, in the literature, is known as the disparity filter (Serrano et al., 2009), which retains all the links that define the relevant structure generated by the weight and the strength assignments with respect to the simple randomness of a null hypothesis. Other types of filter of the same nature exist, and they differ only in the underlying null hypothesis.

8 The use of our definition of collective identity in non-directed graphs is problematic. One should either assume that influence is bidirectional between two connected nodes (in which case, all components of the network must be considered as strongly connected) or it turns impossible to identify any closure.

9 The fact that node “n” is part of the audience for this identity core does not imply that it exclusively receives information from this core, but only for this scope, during this period of time under study, and within this specific network of significant interactions. The same node can be an audience of multiple identities or play different roles at different timescales and scopes.

10 In what follows, we will use the related concept of *k*-core to analyze the cohesion of social identity cores. A *k*-core is the biggest subnetwork where all members of the subnetwork have at least *k* number of ties to other members of the subnetwork. Due to the high computational cost of computing *k*-connectedness in large networks, we will use *k*-cores as a working approximation to *k*-connectedness.

11 This problem is common to empirical sciences and not unlike a neuroscientist dealing with partial neuroimaging recordings or a sociologist dealing with a limited set of survey responses. The datasets that result from limited sampling methods and the models or structures that result from their analysis should not be confused with the object of study but understood as marks or indicators of that object: the underlying social dynamic.

12 An API is a software interface that makes it possible for third parties to request information or operations without directly executing the program or owning or accessing all the data. In our case, Twitter’s API makes it possible, within some limits, to request and receive interaction data between users.

13 This value represents a “standard” among network science researchers in order to consider a community as statistically significant (Ziv et al., 2005).
